# Mutations in immunodeficiency-related genes may increase the risk of infection after CAR-T-cell therapy: a report of two cases

**DOI:** 10.1186/s12879-023-08070-w

**Published:** 2023-02-22

**Authors:** Di Wang, Li He, Chunhui Li, Menglei Xu, Qiuxia Yu, Varlene Daniela Fernandes Almeida, Yimei Que, Yanjie Xu, Yi Xiao, Chunrui Li

**Affiliations:** 1 Department of Hematology, Tongji Hospital, Tongji Medical College, Huazhong University of Science and Technology, 1095 Jie-Fang Avenue, Wuhan, 430030 Hubei China; 2 Department of Hematology, Zhongnan Hospital, Wuhan University, Wuhan, 430071 Hubei China; 3 Tongji Medical College, Huazhong University of Science and Technology, Wuhan, 430030 Hubei China; 4 Immunotherapy Research Center for Hematologic Diseases of Hubei Province, Wuhan, 430030 Hubei China

**Keywords:** CAR-T, Infection, Gene mutations, Sequencing, Immunodeficiency

## Abstract

**Background:**

Chimeric antigen receptor T-cell therapy (CAR-T) has yielded unprecedented efficacy in B-cell malignancies. With the increasing use of CAR-T-cell therapy, infection has become one of the major concerns after CAR-T-cell infusion. Some patients even develop refractory or recurrent infections, posing challenges in treatment, prophylactic, and monitoring strategies. However, the mechanisms underlying the development of these infections were not clear.

**Case presentation:**

We report two cases of infection after CAR-T-cell therapy. Patient 1, diagnosed with multiple myeloma, received anti-B-cell maturation antigen (BCMA) chimeric antigen receptor T (CAR-T)-cell therapy. He developed a refractory urinary infection lasting for over 5 weeks, which was caused by *Candida albicans*. Whole-exome sequencing revealed that he had an IL-17RA gene mutation. Patient 2, diagnosed with acute lymphoblastic B-cell leukaemia, received anti-CD19 and anti-CD22 CAR-T-cell cocktail therapy and remained in complete remission for over 4 years. The patient had pneumonia five times during the 4 years. Whole-exon sequencing revealed that he had a CX3CR1 gene mutation.

**Conclusion:**

For patients who develop persistent or recurrent infections after CAR-T-cell therapy, it is recommended to screen for immunodeficiency-related gene mutations, and the results may contribute to the management of infections post-CAR-T treatment.

## Introduction

Chimeric antigen receptor (CAR) T-cell therapy is a novel therapy representing a paradigm shift in haematological malignancy treatment over the past decade; examples include the use of anti-CD19/22 CAR cocktail therapy to treat relapsed/refractory B-cell lymphoma [1] and anti-B-cell maturation antigen (BCMA) CAR therapy for relapsed/refractory multiple myeloma [2]. Compared with chemotherapy, this novel approach achieves better remission and survival outcomes. However, patients who receive CAR-T-cell therapy often develop immune dysfunction due to multiple factors, making infection one of the most common adverse events after the infusion of CAR-T cells.

Infectious complications of CAR-T cells can occur during the three phases: before CAR-T-cell infusion, immediately after CAR-T-cell infusion, and in long-term follow-up [3]. Infections immediately after CAR-T-cell infusion have been systematically investigated and were predominantly caused by bacteria. Infections caused by other microbes, such as fungi or viruses, are relatively rare. Comparatively, systematic studies focusing on infections in long-term follow-up after CAR-T are limited, especially persistent or repeated infections [4]. In patients receiving CAR-T cells, the cause of the increased susceptibility to refractory or recurrent infection is not clear. Here, we report the detailed infection course in two patients carrying immunodeficiency-related gene mutations, intending to provide a possible explanation.

## Case presentation

**Fig. 1:**
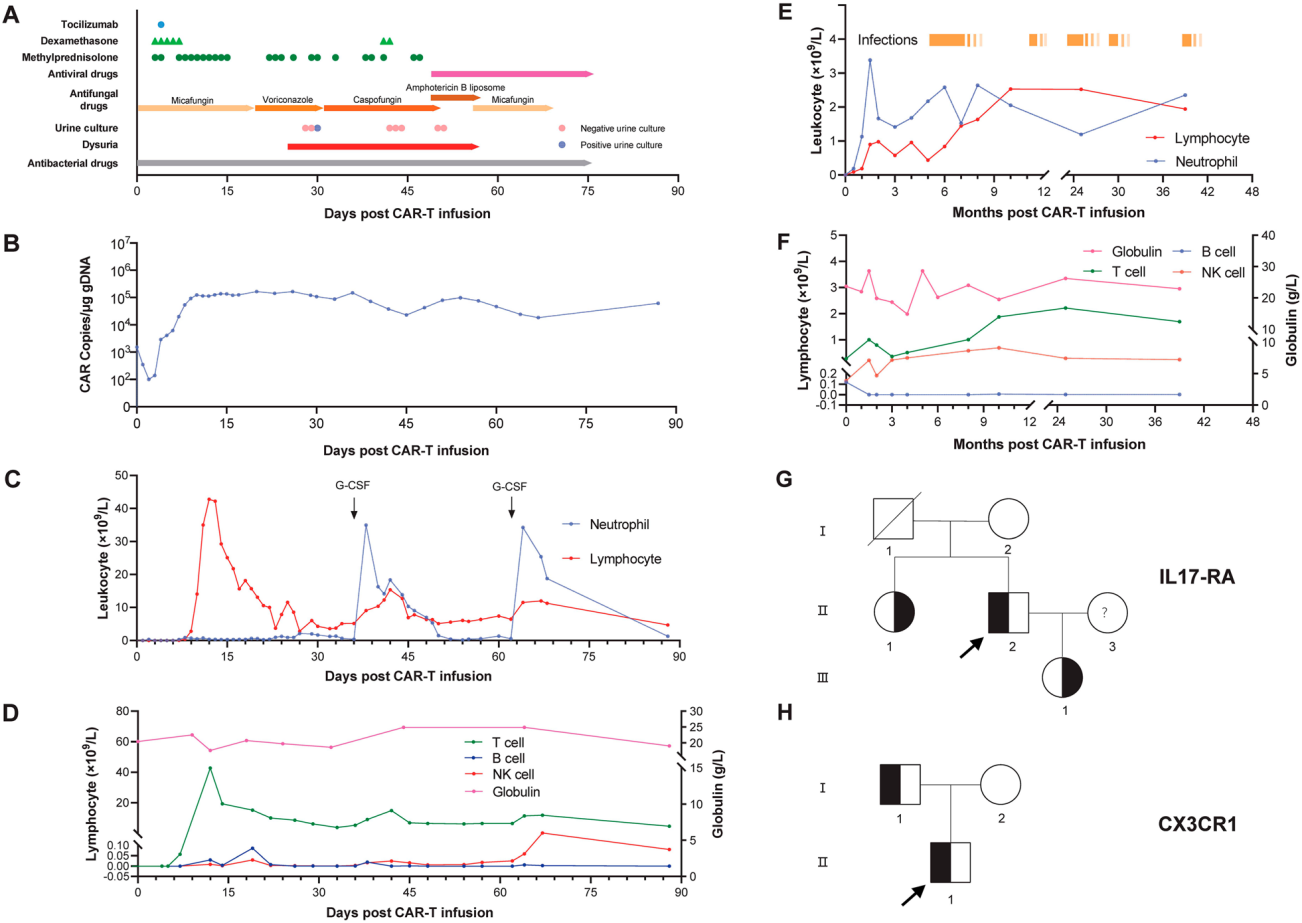
Outline of infections, dynamics of the immune component postinfusion, and mutation pedigree chart of the two patients. **A** Timeline of *Candida albicans* infection in patient one, including the symptom, culture, and drug applied to treat CRS and infection after CAR-T-cell infusion; **B** copy number of CAR transgene in the peripheral blood mononuclear cell of patient one; **C** neutrophil and lymphocyte counts for patient one postinfusion; **D** the serum globulin level and lymphocyte subset counts (T cell, B cell, and NK cell) for patient one; **E** dynamic changes in neutrophil and lymphocyte counts and the duration of each infection for patient two; **F** the serum globulin level and lymphocyte subset counts (T cell, B cell, and NK cell) for patient two; **G** pedigree of gene IL17-RA in the family of patient one (II-2); **H** pedigree of gene CX3CR1 in the family of patient two (II-1). *CAR* chimeric antigen receptor, *G-CSF* granulocyte colony-stimulating factor, *NK* natural killer

Patient 1, a 51-year-old male, was diagnosed with multiple myeloma. He received three lines of previous therapy and was resistant to bortezomib, ixazomib, and lenalidomide. The patient was enrolled in the anti-BCMA CAR-T trial registered on the Chinese Clinical Trial Registry (ChiCTR1800018137). He received lymphodepletion chemotherapy with an FC regimen (fludarabine at 25 mg/m^2^ and cyclophosphamide at 20 mg/kg daily) for 3 consecutive days (days − 4 to − 2) and 1 × 10^6^/kg CAR^+^ cells at Day 0. The amplification of CAR-T cells peaked at Day 13 (Fig. 1B) and remained at a high level for over 2 months. He experienced grade 3 cytokine release syndrome (CRS) for 14 days, which was controlled with tocilizumab and repeated doses of corticosteroids. As shown in Fig. 1A, each dot represents one dose of each drug (8 mg/kg for tocilizumab; 5 mg for dexamethasone; 40 mg for methylprednisolone). He also developed neutropenia and hypogammaglobulinemia (Fig. 1C, D), so prophylactic anti-infective treatment (mainly consisting of antibacterial drugs and antifungal agents including micafungin first and then voriconazole) was given, as well as intravenous immunoglobulin.

The patient complained of dysuria without fever on Day 25, which was considered a urinary tract infection. Empirical antibacterial drugs were given immediately, but the antifungal agent was not changed since we had just downgraded our prophylactic antifungal strategy from micafungin to oral voriconazole. On Day 29, a positive urine culture result indicated *Candida albicans* infection, so antifungal agents were changed from voriconazole to caspofungin the following day. Although urine culture turned negative after caspofungin treatment, the patient’s symptoms were not relieved. Therefore, amphotericin B liposomes were administered and overlapped with caspofungin for 2 days. The symptom of dysuria then gradually resolved; however, the patient developed refractory hypokalaemia even when the dosage of amphotericin B liposomes was reduced. From Day 56, the antifungal agent was changed back to micafungin since the patient had no more complaints (Fig. 1A). To explore the potential reason for persistent refractory fungal infection, whole-exome sequencing was performed. Mutations in the IL-17RA gene (c. 1408 G>A) was revealed, and the mutation abundance was 47.2%. The same mutation was found in his daughter and sister (Fig. 1G).

Patient 2, a 20-year-old male, was diagnosed with acute lymphoblastic B-cell leukaemia 5 years earlier. He received three lines of chemotherapy, including VDCLP and Hyper-CVAD A/B, and he underwent allogenic haematopoietic stem cell transplantation (Allo-HSCT). The patient relapsed 3 months after Allo-HSCT and was admitted to our hospital for CAR-T-cell therapy. Following 3 days of lymphodepletion using fludarabine (25 mg/m^2^) and cyclophosphamide (20 mg/kg), anti-CD19 (2.0 × 10^6^/kg) and anti-CD22 (2.5 × 10^6^/kg) CAR-T-cell cocktail therapy was administered. The patient developed grade 2 CRS for 6 days, along with neutropenia and hypogammaglobulinemia (Fig. 1E, F). Repeated doses of glucocorticoids were employed to control CRS. Antibiotics and intravenous gammaglobulin were given during the period of neutropenia and hypogammaglobulinemia. The patient achieved complete remission at 1 month after CAR-T-cell infusion. However, at the 5th month postinfusion, he was admitted to the hospital for severe pneumonia. Antibiotics and antifungals such as linezolid, tigecycline, meropenem, and voriconazole were given. He was not discharged until 2 months later. Since then, the patient had been hospitalized another four times because of recurrent severe pulmonary infections. All those infections required a long period of antibiotic treatment to be controlled. To explore the potential reason for high susceptibility to infections, whole-exome sequencing was performed. Two mutations were revealed in the gene encoding CX3CR1 (c. 841 G>A and c. 935 C>T), and the mutation abundances were 41.5% and 32%, respectively. The same mutations were found in his father (Fig. 1H).

## Discussion

CAR-T-cell therapy is a promising treatment for relapsed/refractory haematological malignancies, even after three or more lines of chemotherapy. After CAR-T-cell therapy, complications such as cytokine release syndrome (CRS), neurologic toxicities, haematologic toxicities, and infections may occur. In particular, infections not only impair the maintenance of CAR-T efficacy but also lead to repeated hospitalizations and a very large economic burden [5]. Previous reports indicated that the infection rate was approximately 22.6–42% after CAR-T-cell therapy [3, 6]. Park et al. reported that 42% of patients experienced ≥ 1 infection within the first 30 days postinfusion, predominantly bacterial; 31% of patients with complete remission experienced ≥ 1 infection between Days 31 and 180, mainly due to respiratory viruses. The majority of infections were of mild to moderate severity. The infection-related mortality rate was low in these patients, but death sometimes occurred in cases of either multidrug-resistant organisms or polymicrobial infection.

The increased risk of infection in patients undergoing CAR-T-cell therapy is multifactorial [4]. The possible mechanisms include prior multiline treatment, lymphodepleting chemotherapy, and treatment with tocilizumab and steroids leading to unique toxicities. In addition, the on-target-off-tumour effect will cause B-cell aplasia and hypogammaglobulinemia, which further impair the patient’s immunity [7]. Thus, CAR-T-cell immunotherapies pose challenges for acute and long-term infection management. For bacterial infections, antibacterial prophylaxis was not recommended by some studies [8] unless the patient developed fever or infectious symptoms. The risk factors for individuals and the local antibiotic resistance profile should be taken into consideration when empiric antibiotic therapy is administered. For viral infections, prophylactic antiviral therapy, such as oral acyclovir, is essential [9]. For the prevention of fungal infections, some studies suggest prophylaxis using 400 mg/day fluconazole when the neutrophil count is below 500/mm^3^ [8]. In addition, intravenous immunoglobulin replacement is recommended in patients with IgG ≤ 400 mg/dL [10].

However, if CAR-T-cell-treated patients also have immunodeficiency-related gene abnormalities, the accumulated damage to immunity may result in more severe infections. Previous studies have mostly focused on the immune deficiency caused by CAR-T cells, but few studies have focused on the potential immune deficiency caused by gene abnormalities in patients. Our study is the first to use whole-exome sequencing to focus on this aspect. In patient 1, CAR copies remained high for more than 2 months after infusion. At the same time, hypogammaglobulinemia and neutropenia lasted for 3 months. Tocilizumab and repeated doses of corticosteroids were used to control grade 3 CRS. Those factors led to increased risks of infection, as mentioned above. A mutation in the IL-17RA gene, which is essential for mucocutaneous immunity, was revealed in this patient [11]. Gene mutations in molecules in the IL-17 signalling pathway are associated with primary immunodeficiency, which can lead to chronic mucocutaneous candidiasis (CMC). CMC can affect the skin (intertrigo), scalp, mucosal sites (oral thrush; anogenital candidiasis), or nails [12]. Correspondingly, the patient developed a prolonged refractory urinary tract infection. However, neither his daughter nor his sister has a history of infection, even though they bear the same mutation. Therefore, we suspect that the mutation in the IL-17RA gene possibly collaborated with his immunodeficiency and led to the urinary tract infection. However, the patient had many risk factors for infection, such as long-term neutropenia, CAR-T-cell treatment, the use of corticoids and tocilizumab during the period of  CRS, and his primary disease of myeloma. Gene mutation may be only one of the causes of repeated infection in patients.

Patient 2 suffered from repeated severe lung infections spanning the 4 years after CAR-T-cell therapy. Two missense mutations in the CX3CR1 gene were detected. Many key functional aspects of the CX3CR1–CX3CL1 axis, such as the immune response, inflammation, cell adhesion, and chemotaxis, have been identified [13], indicating that mutations may affect host immune function. Similarly, patient 2 had several factors that negatively impacted his immunity: three prior lines of chemotherapy, prior Allo-HSCT, the persistence of B-cell aplasia, and the use of glucocorticoids. In the immunocompromised state of patient 2, mutations in CX3CR1 may further weaken the immune system and consequently result in recurrent severe pneumonia.

Whole-exome sequencing is an efficient technique that can accurately detect gene mutations. The results may predict patients’ risk of developing disease and contribute to personalized medical treatment for patients [14]. Although the mutations we found in the two patients were in a heterozygous state, they may still impair the immune response to some extent and increase susceptibility to recurrent and persistent, even life-threatening, infections after CAR-T-cell therapy. Accordingly, it is possible for other immunodeficiency-related gene defects to cause a higher risk of severe infection. Therefore, for CAR-T-cell-treated patients, especially those who have already experienced severe infections, whole-exome sequencing is recommended to screen for such mutations. If a patient was found to have immunodeficiency-related gene mutations, a longer anti-infection treatment cycle, more prompt detection, higher levels of antibiotics, and additional supportive care may be needed. These patients may also need more frequent monitoring for infections, more aggressive prophylactic strategies, and appropriate and comprehensive patient education, even if B-cell aplasia or hypogammaglobulinemia are resolved [7].

Although this report provided a new aspect of the recognition and management of infections in patients receiving CAR-T-cell therapy, there are still some limitations in our study. First, our evidence is not strong enough to conclude that the refractory/repeated infections of these patients after CAR-T-cell treatment were caused by gene mutation. In addition, we did not fully identify the type of infections or perform a drug sensitivity test to further support our argument. Due to the limited sample size, future large-scale investigations are needed to assess the value of next-generation sequencing for patients who develop persistent or recurrent infections after CAR-T-cell therapy.

## Data Availability

The datasets generated and analysed during the current study are available in the National Center for Biotechnology Information (NCBI) repository, available at https://www.ncbi.nlm.nih.gov/sra/PRJNA924003.

## Abbreviations

VDCLP: Vincristine, daunorubicin, cyclophosphamide, L-asparaginase and prednisone
Hyper-CVAD A: Cyclophosphamide, vincristine, adriamycin, and dexamethasone
Hyper-CVAD B: Methotrexate and high-dose cytarabine
